# Protocol for a Virtual Life Story Club to Address Apathy and Loneliness in Older Adults

**DOI:** 10.1093/geroni/igaf122.3405

**Published:** 2025-12-31

**Authors:** Atul Bhattiprolu, Eunah Song, Sophie Anfang, Samantha Yip, Lily Zhou, Jennifer Wong, Ying Jin, Mirnova Ceïde

**Affiliations:** Albert Einstein College of Medicine, Bronx, New York, United States; Albert Einstein College of Medicine, Bronx, New York, United States; Albert Einstein College of Medicine, Bronx, New York, United States; Albert Einstein College of Medicine, Bronx, New York, United States; Life Story Club, Brooklyn, New York, United States; Life Story Club, Brooklyn, New York, United States; Albert Einstein College of Medicine, Bronx, New York, United States; Albert Einstein College of Medicine, Bronx, New York, United States

## Abstract

Life Story Club (LSC) offers storytelling groups as virtual reminiscence therapy (vRT) to help older adults reduce loneliness and build social connections. Apathy and loneliness are significant modifiable risk factors for dementia, contributing to cognitive decline and emotional distress. This mixed-methods feasibility study evaluates the impact of LSC’s vRT on apathy and loneliness in older adults, aged 60+ and without dementia, who are at risk for social isolation. Participants (n = 18) were recruited from community organizations, with inclusion criteria requiring self-reported loneliness. vRT sessions were conducted in English and Spanish to enhance accessibility for Bronx residents. Apathy was measured using the GDS3A, a subset of the Geriatric Depression Scale, and loneliness was assessed with the UCLA Loneliness Scale. Qualitative interviews were also used to assess participant satisfaction and the perceived impact of vRT on social connections. Data were collected at baseline, with follow-up 3 months later. Follow-up data were available for 11 participants regarding apathy, 5 of whom had scores demonstrating apathy at baseline, and 10 participants regarding loneliness. In the initial phase of the pilot, 60% of participants showed stable or improved loneliness scores, whereas 64% showed stable or improved apathy scores, at 3 months compared to baseline. Participants also reported that vRT facilitated meaningful social connections, with one group organizing two in-person events. Further analysis of participants’ experiences will refine the LSC protocol, informing future randomized controlled trials and potentially providing a scalable intervention to address cognitive decline, loneliness, and social isolation in the Bronx community.

